# Risk stratification of new-onset psychiatric disorders using clinically distinct traumatic brain injury phenotypes

**DOI:** 10.1186/s13690-024-01346-w

**Published:** 2024-08-02

**Authors:** Nelofar Kureshi, Abraham Nunes, Cindy Feng, David B. Clarke, Syed Sibte Raza Abidi

**Affiliations:** 1 Department of Surgery (Neurosurgery), Dalhousie University, Halifax, NS Canada; 2 Department of Psychiatry, Dalhousie University, Halifax, NS Canada; 3 Department of Community Health and Epidemiology, Dalhousie University, Halifax, NS Canada; 4 Brain Repair Centre, Dalhousie University, Halifax, NS Canada; 5 Faculty of Computer Science, Dalhousie University, Halifax, NS Canada

**Keywords:** Traumatic brain injury, New-onset psychiatric disorders, Latent class analysis, Public health

## Abstract

**Background:**

Patients with traumatic brain injury (TBI) constitute a highly heterogeneous population, with varying risks for New-onset Psychiatric Disorders (NPDs). The objectives of this study were to identify TBI phenotypes and determine how NPDs differ among these phenotypes.

**Methods:**

Hospitalized TBI patients from 2003 to 2019 were obtained from the provincial trauma registry. Propensity score matching was conducted to balance covariates among patients with TBI and controls. To uncover heterogeneity in TBI, latent class analysis (LCA)-based clustering was applied. LCA was conducted separately for two TBI cohorts: those with and without pre-injury psychiatric conditions The effect of classes on NPDs was assessed using log binomial regression models.

**Results:**

A total of 3,453 patients with TBI and 13,112 controls were included in the analysis. In a conditional regression involving propensity matched patients with TBI and controls, TBI was significantly associated with the development of NPD-A (OR: 2.78; 95% CI: 2.49–3.09), as well as NPD-P (OR: 2.36; 95% CI: 2.07–2.70). Eight distinct latent classes were identified which differed in the incidence of NPDs. Four classes displayed a 53% (RR:1.53; 95% CI: 1.31–1.78), 48% (RR:1.48; 95% CI: 1.26–1.74), 28% (RR:1.28; 95% CI: 1.08–1.54), and 20% (RR: 1.20, 95%CI: 1.03–1.39), increased NPD risk.

**Conclusion:**

TBI is a significant predictor of NPDs. There are clinically distinguishable phenotypes with different patterns of NPD risk among patients with TBI. Identifying individuals with respect to their phenotype may improve risk stratification of patients with TBI and promote early intervention for psychiatric care in this vulnerable population.

**Supplementary Information:**

The online version contains supplementary material available at 10.1186/s13690-024-01346-w.

**Table:** 

Text box 1. Contributions to the Literature
• Patients with TBI have increased odds of New-onset Psychiatric Disorders (NPDs) compared with population-matched controls.
• TBI is a heterogenous disorder with clinically distinct phenotypes arising from demographic, injury variables, and pre-injury psychiatric history.
• Prolonged hospital stays and discharge to supportive care may indicate functional and cognitive decline after injury and are associated with an elevated NPD risk.
• In patients with pre-injury psychiatric disorders, psychiatric burden is associated with the incidence of NPDs.
• A risk stratification approach using latent class analysis resulting in clustering of patients into phenotypes may be effective in identifying TBI patients who are likely to require early intervention for NPDs.

## Introduction

Traumatic brain injury (TBI) is a significant contributor to global injury burden, with an age-standardized incidence rate of 369 per 100,000 population [1]. Each year in Canada, there are approximately 166,455 TBIs [2], resulting in roughly 20,000 hospitalizations and annual direct medical costs of $120.7 million CAD [3]. TBI is a leading cause of mortality in Canada, accounting for approximately 23% of all injury-related deaths [2].

New-onset psychiatric disorders (NPDs) are common after TBI [4]. Factors such as the location of brain lesion, prior lifetime psychiatric disorders, family function, family psychiatric history, and socioeconomic status have shown significant associations with the development of NPDs following pediatric TBI [5–9]. Furthermore, NPDs are more common in adults with a history of pediatric TBI compared with controls without childhood head injuries, and the presence pre-injury lifetime psychiatric disorders notably predicts the occurrence of NPDs [10].

Despite the growing body of research on the link between TBI and NPDs [4], there remain significant gaps in our knowledge. Pre-injury psychiatric history is a well-established risk factor for NPDs in patients with TBI [7, 10, 11]. Several studies have explored the risk of NPDs in patients without a prior psychiatric history, finding that many such patients also develop post-injury disorders [12, 13]. Yet few studies have compared the risk of NPDs in TBI patients with and without pre-injury psychiatric disorders. Secondly, TBI patients constitute a highly heterogeneous population, with different profiles of risk and long-term outcomes [14–16]. While most studies have examined the relationship between various predictors and NPDs following TBI, none have explored the heterogeneity in the relationship between demographics, injury, psychiatric history and NPDs in patients with TBI.

The current study addresses these knowledge gaps by defining NPD as two distinct outcomes experienced by those with and without a psychiatric history. A large population-based dataset of patients with TBI and controls was used to demonstrate the difference in the development of NPDs over two years. Using demographic, injury, and clinical history data, clustering models were specified and fitted to identify phenotypes of patients at high risk of NPDs. We hypothesized that this approach could identify patient clusters, each with a distinct profile of clinical characteristics associated with significant differences in NPDs.

This investigation addressed three specific research questions: (1) What is the effect of TBI on the development of NPDs in a propensity-matched sample of injured patients and population-based controls? (2) Within the TBI cohort, are there specific phenotypes which differ with respect to demographics, injury variables, and pre-injury psychiatric conditions? (3) Are these phenotypes associated with the development of NPDs?

## Methods

### Data sources

#### Nova Scotia Trauma Registry (NSTR)

The TBI cohort was obtained from the NSTR, a comprehensive, provincial, population-based trauma registry. NSTR is an extensive database that captures demographic and clinical data on all major trauma cases requiring hospitalization in the province.

#### Canadian Institute of Health Information- Discharge Abstract Database (CIHI DAD)

CIHI DAD captures administrative, clinical and demographic information on hospital discharges (including deaths, sign-outs and transfers). Psychiatric diagnoses were based on the International Classification of Diseases, Tenth Revision (ICD-10) codes F04-F99.

#### MSI Physician Billing (MED)

MED records physician billing information with details of a service encounter between an individual and a provider. Psychiatric diagnoses were based on the International Classification of Diseases, Ninth Revision (ICD-9) codes 210–319.

#### National Ambulatory Care Reporting System (NACRS)

NACRS contains data for all hospital-based and community-based ambulatory care. Psychiatric diagnoses were based on ICD-10 codes F04-F99.

## Study setting and participants

### Exposure

The exposure of interest in this study was the occurrence of TBI. We included patients with TBI admitted to a provincial hospital between 2003 and 2019. The severity of TBI was determined using the Abbreviated Injury Scale (AIS). Patients presenting solely with scalp abrasions or superficial lacerations (not representative of intracranial injury), are often categorized as AIS Head score = 1 [17], and were excluded from the study cohort. Only subjects with a maximum AIS Head score ≥ 2 were included in the study. This definition for TBI severity includes a broad range of injury severity and has been used in previous studies [17–20]. Controls were obtained from the provincial health insured registry and did not have a history of TBI or develop TBI in the two years of follow up based on clinical encounters (See Supplementary Data).

### Covariates

Guided by the literature, age, sex, medical comorbidities (defined through the Charlson Comorbidity Index [CCI]), pre-injury psychiatric diagnoses, AIS Head score, injury mechanism, discharge destination and length of stay (LOS) in acute care facility were identified as important covariates to consider as potential confounders.

### Outcome

The outcome of interest was NPD in the first two years post-TBI. Previous studies have defined NPD (also referred to as novel psychiatric disorder) to occur in one of two conditions [6–8]: (1) the development of a psychiatric disorder after injury in a patient with no lifetime preinjury psychiatric disorder; or (2) the development of a post-injury psychiatric disorder in a patient with a lifetime psychiatric disorder that was never before present (e.g., a subject with a lifetime history of major depressive disorder who develops anxiety disorder after the injury would receive the classification, but would not if only a new episode of major depression occurred). In many cases, the “lifetime psychiatric disorder” variable refers to any psychiatric disorder present prior to the injury [10, 13]. In the current study, we defined NPD as two distinct outcomes, namely, NPD in the *absence* of psychiatric history (NPD-A) and NPD in the *presence* of psychiatric history (NPD-P). These outcomes were defined to understand influence of pre-injury psychiatric history on the occurrence of NPDs.

### Study design

**Fig. 1:**
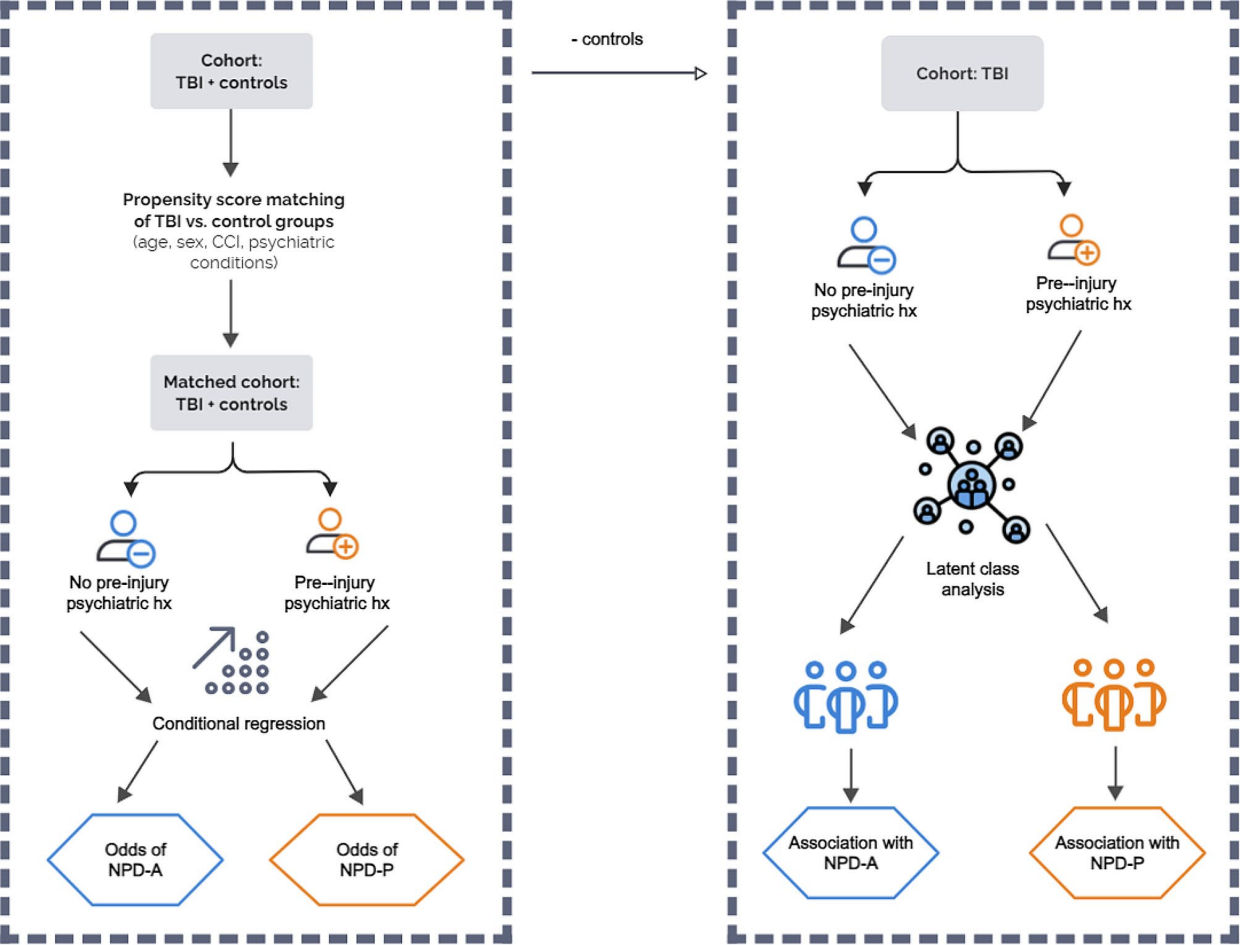
Study design. Propensity score matching of TBI vs. control groups (left panel). LCA for two TBI cohorts: those with and without pre-injury psychiatric conditions (right panel)

The overall study design is presented in Fig. 1. The study is a matched cohort design with a two-year follow-up for outcome collection. The study unfolded in two phases: firstly, we assessed the development of NPDs in a propensity-matched cohort of patients with TBI and population-based controls; secondly latent class analysis (LCA) was applied to the TBI cohort, identifying TBI phenotypes.

## Data analysis

### Propensity score matching

The covariates entered into the propensity score were age, sex, CCI group (no comorbidities [0], mild [1-2], moderate [3-4], and severe [≥ 5]), and psychiatric diagnoses (ICD9:290–319, ICD10: F04-F99). Cases and controls were matched using greedy nearest neighbor matching [21] without replacement using a calliper width of 0.2 standard deviations of the logit of the propensity score. A matching ratio of 4-to-1 (up to 4 matches of controls per case) was used (See Supplementary Data). The matched cohort was then divided into those with and without pre-injury psychiatric conditions before further analysis.

### Latent class analysis

LCA was used to derive clinical phenotypes of patients with TBI, which represent their distinct underlying individual attributes. Details on the LCA methodology are described in the Supplementary methods. Since pre-injury psychiatric disorders are common among individuals with TBI, we aimed to study the influence of TBI on those with existing psychiatric disorders as well as those without pre-injury psychiatric conditions; LCA was conducted separately for these two cohorts. Confirmation of ICD codes related to psychiatric conditions in the two years prior to the injury were used to define pre-injury psychiatric conditions. For those without psychiatric history, indicators included sex, categories of age, categories of CCI, injury mechanism, injury severity (maximum AIS Head), prolonged LOS, and discharge destination (Table 1). For those with pre-injury psychiatric disorders, we used the same indicators and added past psychiatric conditions as additional indicators. Only psychiatric conditions with a prevalence of > 10% were included: disorders relating to sleep, organic conditions, mood, anxiety, drug substance abuse disorder (SUD), and alcohol SUD. For age, we utilized four categories representing different stages of life: pediatric (0–18 years), young adult (19–35 years), middle-aged adult (36–59), and older adult (≥ 60 years). LOS was dichotomized as brief or prolonged, defined as ≥ 21 days based on previous research [22, 23]. Relative fit was evaluated using the Bayesian Information Criterion (BIC), the sample-size adjusted BIC (SABIC), and Vuong-Lo-Mendell-Rubin adjusted likelihood ratio test (VLMR-LRT) [24]. Empirical robustness was complemented by interpretability during the process of model selection [25].

**Table 1:** Domains, descriptions, and data sources of indicators used in LCA modeling

Domain	Indicator	Description	Data source
Demographics	Sex	Sex of patient, defined as male/female	NSTR^1^
	Age	Categorized as pediatric (0–18 years), young adult (19–35 years), middle-aged adult (36–59), and older adult (≥ 60 years).	NSTR
Medical history	Medical comorbidities	Charlson comorbidity index score categorized as no comorbidities (0), mild (1–2), moderate (3–4), or severe (≥ 5).	HDNS^2^
	Pre-injury psychiatric conditions^3^	Sleep disorders, Organic disorders, mood disorders, anxiety disorders, drug-related substance abuse, alcohol-related substance abuse. Comorbid psychiatric conditions were defined as > 1 pre-injury psychiatric conditions.	HDNS
Injury-related variables	Injury mechanism	Categorized as falls, motor vehicle collisions (MVC), violence, or other	NSTR
	Injury severity	Defined by the maximum Abbreviated Injury Score (AIS). AIS Head between 2–5 were considered as categorical variables	NSTR
	Length of stay	Numbers of days at the acute care facility. Categorized as prolonged if LOS ≥ 21 days.	NSTR
	Discharge destination	The destination of the patient upon discharge from the acute care facility. Categorized as another acute care facility, chronic care facility, nursing home, rehabilitation facility, home with support services, or home.	NSTR

^1^ NSTR: Nova Scotia Trauma Registry

^2^ HDNS: Health Data Nova Scotia

^3^ Only psychiatric conditions with a prevalence of > 10% were included in LCA. The full list of psychiatric disorder categories is presented in Supplementary Table 1

Characteristics were compared across the identified phenotypes. Categorical variables were reported as number (percentage) and were compared across classes using the chi square test. In the overall matched cohort, conditional regression was used to account for the matched sets and to provide odds ratios (OR) as the measure of association between the exposure (TBI) and the outcome (NPD) [26]. Following the identification of latent classes, a conditional logistic regression analysis was also conducted within each phenotype to compare the odds of NPD in TBI patients with their matched controls. The OR from conditional regression estimates the relative odds of NPD occurring in the TBI group compared to the control group, while controlling for the matching variables. Log-binomial regression models were fitted to observe the association between phenotypes and the outcome of NPD. We used log-binomial regression to directly estimate the relative risk (RR), which is more interpretable than the OR when the outcome prevalence is high (commonly defined as > 10%) [27]. A *p* < 0.05 was considered as statistical significance.

## Results

### Characteristics of study population

There were 3,453 patients with TBI and 13,112 matched controls in the analysis. Table 2 shows the frequencies for the matched variables between cases and controls. TBI and control groups did not significantly differ in age, sex, comorbidities, or history of psychiatric disorders. In the two-year follow-up, a significantly greater proportion of TBI patients developed NPD-A (35.6% vs. 16.9%, *p* < 0.001) and NPD-P (46.9% vs. 27.7%, *p* < 0.001) than matched controls. Psychiatric diagnostic categories are described in Supplementary Table 1.

**Table 2:** Characteristics of the matched cohort

Characteristic	TBI *N* = 3,453	Control *N* = 13,112	*P*
Age (mean, SD)	51.21 ± 25	51.66 ± 25	0.36
Sex			
Male (n, %)	2,430 (70.4)	9,121(69.6)	0.37
Female (n, %)	1,023 (29.6)	3,991 (30.4)	
Charlson comorbidity index			
None (n, %)	2,026 (58.7)	7,476 (57.0)	0.41
Mild (n, %)	976 (28.3)	3,892 (29.7)	
Moderate (n, %)	277 (8.0)	1,085 )8.3)	
Severe (n, %)	174 (5.0)	659 (5.0)	
Psychiatric history			
None (n, %)	2,206 (63.9)	8,275 (63.1)	0.41
Any (n, %)	1,247 (36.1)	4,837 (36.9)	
NPD-A^1^	786 (35.6)	1,399 (16.9)	< 0.001
NPD-P^2^	585 (46.9)	1,339 (27.7)	< 0.001
Injury type^3^			
Blunt (n, %)	3,397 (98.4)	-	
Penetrating (n, %)	51 (1.5)	-	

^1^ All patients within the outcome of NPD-A, did not have a documented history of psychiatric disorders in the two years prior to injury

^2^ All patients within the outcome of NPD-P had a documented history of psychiatric disorders in the two years prior to injury

^3^ A small proportion of injuries were due other mechanisms including burns, drowning, or asphyxia

### Comparison of TBI vs. non-TBI patients for NPDs

In conditional regression analysis performed on the matched groups in the overall study population (Table 3), TBI was significantly associated with the development of NPD-A (OR: 2.78; 95% CI: 2.49–3.09), as well as NPD-P (OR: 2.36; 95% CI: 2.07–2.70).

**Table 3:** Results of the conditional logistic regression with adjusted odds ratio (OR) and 95%confidence interval (CI) for NPDs in the matched cohort

	NPD-A^1^			NPD-*P*^2^		
	Odds ratio^3^	95%CI	*p*	Odds ratio^4^	95%CI	*P*
Control	REF			REF		
TBI	2.78	2.49–3.09	< 0.001	2.36	2.07–2.70	< 0.001

^1^ All patients within the outcome of NPD-A, did not have a documented history of psychiatric disorders in the two years prior to injury

^2^ All patients within the outcome of NPD-P had a documented history of psychiatric disorders in the two years prior to injury

^3^ Adjusted for matching on age, sex, and comorbidities

^4^ Adjusted for matching on age, sex, comorbidities, and psychiatric history

REF: reference level

### Latent class analysis

Table 4 presents fit indices for latent class models ranging from one to five classes. A 4-class solution provided the best fit for both TBI cohorts. Supplementary Tables 2–3 provide the mean posterior probabilities of the selected models, representing the average likelihood of individuals being assigned to their respective latent classes. All class assignments were > 80%, demonstrating high class assignment certainty for individuals.

**Table 4:** Fit statistics for latent class analysis

	TBI cohort without pre-injury psychiatric disorders				
Model	AIC^1^	BIC^2^	SABIC^3^	LR^4^	LRT^5^
2 class	27026.67	27248.66	26952.52	2511.56	
3 class	26290.58	26626.42	26176.43	1841.49	< 0.001
4 class*	26131.58	26581.26	25977.43	1693.07	< 0.001
5 class	26077.50	26641.01	25883.34	1594.64	< 0.001
	**TBI cohort with pre-injury psychiatric disorders**				
2 class	24702.05	24973.73	24599.62	6570.99	
3 class	24317.83	24727.92	24161.40	6192.17	< 0.001
4 class*	24160.17	24708.67	23949.74	6077.18	< 0.001
5 class	24016.84	24703.73	23752.40	5870.54	< 0.001

^1^ Akaike information criterion

^2^ Bayesian information criterion

^3^ Sample-size adjusted BIC

^4^ Likelihood ratio G^2^ statistic

^5^ Vuong-Lo-Mendell-Rubin adjusted likelihood ratio test

*The SABIC and LRT indicated the 5-class model as the most appropriate. Lower values for BIC and SABIC indicate relatively better balance between parsimony and model fit. Emphasis was placed on BIC, SABIC, and VLMR-LRT given evidence showing their unique strength in identifying the ideal number of classes. The 4-class model provided a more clearly defined representation of the data over the 5-class models. For these reasons, the 4-class model was selected

### Class descriptions for TBI cohort without pre-injury psychiatric conditions

**Fig. 2:**
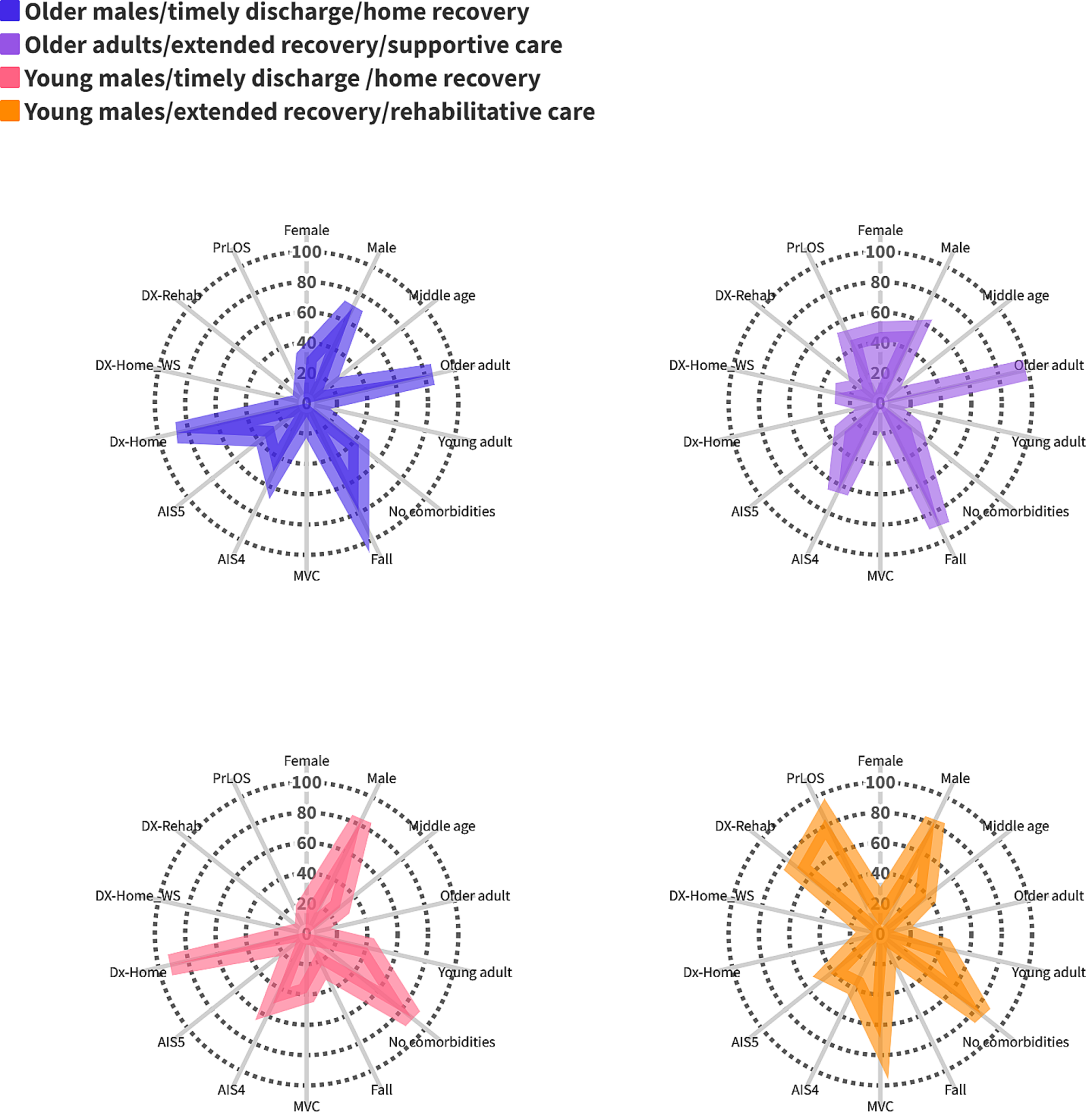
Radar plots for four phenotypes of TBI patients without psychiatric conditions

Table 5 summarizes the descriptive statistics of the four classes and a visual representation is shown in Fig. 2. Significant differences were observed across all characteristics between classes. Additionally, the incidence of NPD-A showed a significant variation between classes (*p* < 0.001).

**Table 5:** Analysis of patient characteristics by latent class for the TBI cohort without pre-injury psychiatric conditions. Adjusted relative risk (RR) of NPD-A is estimated from log binomial regression model. Odds ratio (OR) comparing patients with TBI to their matched controls is estimated using conditional regression for matched subjects

Indicator	Older males/ timely discharge/ home recovery *n* = 646	Older adults/ extended recovery/ supportive care *n* = 376	Young males/ timely discharge /home recovery *n* = 854	Young males/ extended recovery/ rehabilitative care *n* = 315	*P*
	Percentage^1^				
Sex					
Female	30	47	17	18	< 0.001
Male	70	53	83	82	
Age					
Older adult	85	98	0.6	9	< 0.001
Middle-aged adult	14	2	28	38	
Young adult	-	-	41	42	
Pediatric	0.9	-	31	12	
Charlson Comorbidity					
None	44	26	88	85	< 0.001
Mild	42	39	12	15	
Moderate	10	19	-	-	
Severe	4	17	-	-	
Mechanism					
Fall	79	89	23	10	< 0.001
MVC	9	3	39	69	
Violence	-	2	15	5	
Other	11	5	23	16	
AIS Head					
2	3	3	18	4	< 0.001
3	12	3	23	16	
4	50	64	51	38	
5	35	30	9	42	
Discharge status					
Another acute care facility	13	43	5	13	< 0.001
Chronic care facility		2	-	-	
Home	87	2	92	10	
Home with support	-	29	3	6	
Nursing home	-	10	-	-	
Rehabilitation facility	-	15	-	70	
Prolonged LOS					
Yes	5	46	2	84	< 0.001
No	95	55	98	17	
NPD-A^2^	35	45	29	43	< 0.001
Relative risk (95%CI)	1.20	1.53	REF	1.48	
	(1.03–1.39*)	(1.31–1.78*)	REF	(1.26–1.74*)	
Odds ratio (95%CI)	2.69 (2.19–3.32*)	3.46 (2.76–4.34*)	2.11 (1.75–2.54*)	3.96 (2.91–5.37*)	

^1^Suppression of cell counts: If any cell is < 5, the value (and its corresponding percentage) is suppressed and indicated with dash (-). If a cell is < 5 and only one value is suppressed in a row or column, the next highest value in that row or column is also suppressed

^2^ NPD-A was not used as an indicator in LCA. Differences in NPD-A incidence were determined after classes were modeled

REF: reference level

*p-value < 0.05

#### Older males/timely discharge/home recovery

This class was composed largely of older adult (85%) males (70%) who experienced falls (79%). They were discharged home (87%) following a brief LOS (95%).

#### Older adults/extended recovery/supportive care class

This class was composed largely of older adults (98%) who experienced falls (89%). They were discharged to other acute care facilities (43%) or home with support services (29%) after a prolonged LOS (46%).

#### Young males/timely discharge /home recovery class

This class was composed of young adult (41%) males (83%) whose injuries were caused by MVCs (39%). These individuals were discharged home (92%) after a brief LOS (98%).

#### Young males/extended recovery/rehabilitative care class

Members of this class were young adult (42%) and middle age (38%) males (82%). Their head injury was mostly caused by MVCs (69%) and resulted in a severe injury (AIS = 5; 42%). The majority of this class had a high probability of being discharged to a rehabilitation facility (70%) after a prolonged LOS (84%).

Within each of the identified phenotypes, the odds of NPD-A were significantly higher among TBI patients than their matched controls. The risk ratio of NPD-A in the *older adult/extended recovery/supportive care* class was 53% higher than the *young males/timely discharge/home recovery* class (RR:1.53; 95% CI: 1.31–1.78, Table 5). Additionally, the *young males/extended recovery/rehabilitative care* class exhibited a 48% higher RR (RR:1.48; 95% CI: 1.26–1.74) and the *older males/timely discharge/home* recovery class demonstrated a 20% higher RR (RR: 1.20, 95%CI: 1.03–1.39) than the *young males/timely discharge/home recovery* class.

### Class descriptions for TBI cohort with pre-injury psychiatric conditions

**Fig. 3:**
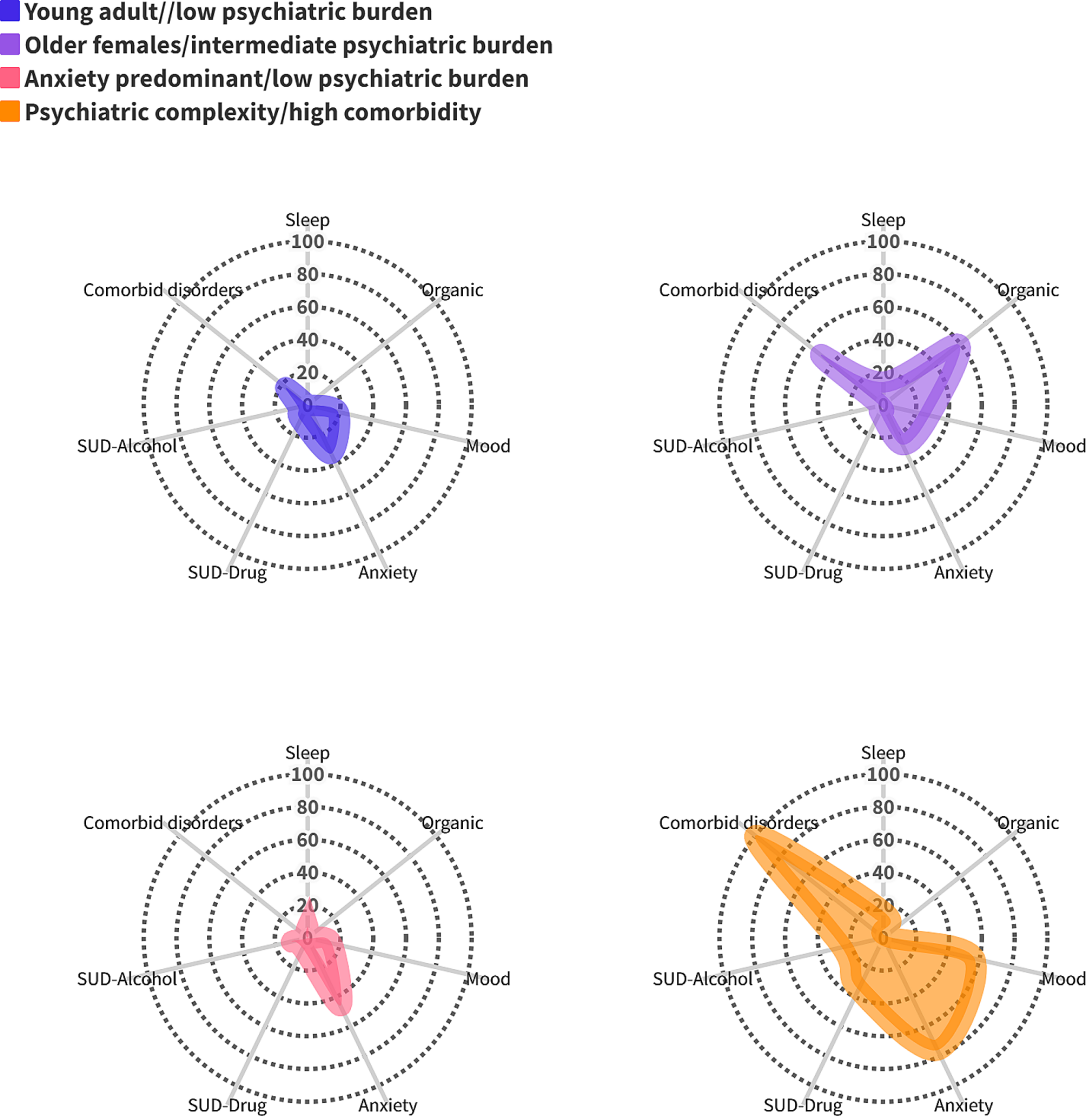
Radar plots for pre-injury psychiatric burden for four phenotypes of TBI patients with psychiatric conditions

Table 6 summarizes the estimated profiles of the 4-class model for the TBI cohort with pre-injury psychiatric conditions and a visual representation is presented in Fig. 3. Significant differences were observed across all indicators between classes. Moreover, the incidence of NPD-P showed a significant variation between classes (*p* = 0.02). The distinct classes identified in this cohort are as follows:

**Table 6:** Analysis of patient characteristics by latent class for the TBI cohort with pre-injury psychiatric conditions. Adjusted relative risk (RR) of NPD-P is estimated from log binomial regression model. Odds ratio (OR) comparing patients with TBI to their matched controls is estimated using conditional regression for matched subjects

Indicator	Young adult//low psychiatric burden *N* = 271	Older females/intermediate psychiatric burden *N* = 287	Anxiety predominant/ low psychiatric burden *N* = 356	Psychiatric complexity/high comorbidity *N* = 330	*P*
	Percentage^1^				
Sex					
Female	20	60	31	33	< 0.001
Male	80	40	69	67	
Age					
Older adult	-	98	53	25	< 0.001
Middle-aged adult	-	2	46	52	
Young adult	49	-	-	-	
Pediatric	34	-	-	-	
Charlson Comorbidity					< 0.001
None	85	7	48	58	
Mild	16	44	37	34	
Moderate	-	31	9	5	
Severe	-	18	6	4	
Mechanism					
Fall	11	94	71	42	< 0.001
MVC	57	-	10	23	
Violence	17	-	7	16	
Other	15	4	12	19	
AIS Head					
2	15	-	6	9	< 0.001
3	22	-	14	22	
4	45	67	59	47	
5	19	27	20	21	
Discharge status					
Another acute care facility	8	23	21	18	< 0.001
Chronic care facility	-	3	-	-	
Home	67	18	64	59	
Home with support	5	23	7	5	
Nursing home	-	30	-	-	
Rehabilitation facility	21	4	7	16	
Prolonged LOS					
Yes	28	40	15	32	< 0.001
No	72	60	85	68	
Sleep disorders	-	14	7	16	< 0.001
Organic disorders	-	59	-	2	< 0.001
Mood disorders	19	29	15	57	< 0.001
Anxiety disorders	33	27	46	76	< 0.001
Drug abuse disorder	10	5	7	35	< 0.001
Alcohol abuse disorder	6	2	10	26	< 0.001
Comorbid psychiatric disorders	17	49	-	100	< 0.001
NPD-P^2^	41	44	49	53	0.02
Relative risk (95%CI)	REF REF	1.07 (0.88–1.30)	1.19 (1.00-1.43)	1.28 (1.08–1.54*)	
Odds ratio (95%CI)	2.62 (2.08–3.31*)	2.21 (1.73–2.82*)	2.57 (1.77–3.72*)	2.13 (1.63–2.78*)	

^1^Suppression of cell counts: If any cell is < 5, the value (and its corresponding percentage) is suppressed and indicated with dash (-). If a cell is < 5 and only one value is suppressed in a row or column, the next highest value in that row or column is also suppressed

^2^ NPD-P was not used as an indicator in LCA. Differences in NPD-P incidence were determined after classes were modeled

REF: reference level

*p-value < 0.05

#### Young adult/low psychiatric burden class

Individuals in this class were mostly young adult (49%) males (82%). MVCs were the major cause of TBI (57%). These individuals were discharged home (67%) after a brief LOS (72%). This class had low probabilities of pre-injury psychiatric conditions (mood disorders [19%], anxiety disorders [33%], drug SUD [10%], alcohol SUD [6%]), and low probabilities of comorbid psychiatric disorders (17%).

#### Older females/intermediate psychiatric burden class

Members of this class were older (98%) females (60%) with a high probability of falls as the cause of TBI (94%). This class was discharged home with support services (23%) or to a nursing home (30%). These individuals had pre-injury organic disorders (59%), mood disorders (29%), anxiety (27%), and a 49% probability of comorbid conditions.

#### Anxiety predominant/low psychiatric burden class

This class was composed of middle-age (46%) and older adult (53%) males (69%) who experienced falls (71%). They were discharged home (64%). Pre-injury anxiety (46%) was the most common disorder, but there were but no comorbid disorders in this class.

#### Psychiatric complexity/high comorbidity class

This class was composed of middle-aged (52%) males (67%), who experienced falls (42%) and were discharged home (59%). This class had the highest probabilities of pre-injury psychiatric disorders: sleep (16%), mood (57%), anxiety (76%), drug-related substance abuse (35%), alcohol-related substance abuse, (26%), and the highest probability of comorbid conditions (100%).

Within each of the identified phenotypes, the odds of NPD-P were significantly higher among TBI patients than their matched controls. The *psychiatric complexity/high comorbidity* class exhibited a 28% higher risk (RR:1.28; 95% CI: 1.08–1.54) of NPD-P compared to the *young adult/low psychiatric burden* class (Table 6).

## Discussion

In the present study, a matched cohort was used to determine the increased risk of NPDs in those with and without TBI. Additionally, the potential effects of demographics, injury variables, medical comorbidities, and pre-injury psychiatric conditions on the NPDs experienced by TBI patients were described. We established that TBI is a heterogenous population demonstrating distinct clinical profiles (phenotypes), that show significant differences in the development of NPDs, suggesting their potential utility in risk stratification. This study is a proof-of-concept and further research is needed to assess the utility of these phenotypes to guide clinical management of TBI patients.

Previous research indicates that patients with TBI are at risk for developing NPDs with rates ranging from 18.3 to 60.8% in the first year post-injury [11, 28]. Our results are consistent with these findings as the rates of NPD-A and NPD-P at two years in the current study were between 36 and 47%. Results from a conditional regression model in the matched cohort revealed that in the cohort without pre-injury psychiatric conditions, the odds of developing NPD were 2.8 times greater in those with TBI. In the cohort with pre-injury psychiatric conditions, those with TBI had 2.4 greater odds for developing NPD compared to controls. Thus, head injury alone and in combination with pre-injury psychiatric conditions appears to contribute to increased risk of post-injury psychiatric disorders compared to controls. A previous population-based study demonstrated an increase in risk for all psychiatric outcomes after head injury in patients without pre-injury psychiatric history [29]. Prior studies also support the finding that among TBI patients, pre-injury disorders are significant predictors of post-injury disorders [12, 30].

We speculate that there are several justifications for how TBI may act independently and in conjunction with pre-injury psychiatric disorders to increase the risk of NPDs. These mechanisms involve an interplay of disrupted connectivity, neuroinflammation, neurochemical imbalances, psychosocial factors, and environmental influences. Firstly, the neuroinflammatory response triggered by TBI can persist long after the initial injury. Chronic neuroinflammation has been linked to alterations in brain function and structure, affecting regions critical for emotional regulation, such as the prefrontal cortex and the limbic system, thereby contributing to the development and exacerbation of psychiatric disorders [31]. Additionally, TBI affects the brain’s structural and functional connectivity, leading to impairments in the communication between different brain regions involved in mood regulation, cognition, and behavior [32]. Disrupted connectivity in the default mode network, salience network, and fronto-limbic circuits has been associated with various psychiatric disorders, including depression, bipolar disorder, schizophrenia, autism, and PTSD [33]. TBI can lead to imbalances in neurotransmitters such as serotonin, dopamine, and glutamate, which play crucial roles in mood regulation, and their disruption can contribute to the development of various psychiatric disorders. Furthermore, the psychological distress of sustaining a TBI, such as loss of independence, changes in social roles, and difficulties with daily activities, may contribute to the development of psychiatric disorders like depression, anxiety, and PTSD [34]. Finally, pre-existing genetic vulnerabilities and environmental factors, including stress, trauma, and lack of social support, may interact with the neurobiological changes caused by TBI, increasing the risk of manifestation of psychiatric conditions [4].

Among TBI patients without any pre-injury psychiatric history, the emerging phenotype was predominately determined by LOS and discharge destination while accounting for the effects of age-related vulnerability to psychiatric disorders. The *young males/timely discharge/home recovery* phenotype was characterized by a notable proportion of young adults and pediatric patients and showed the lowest NPD-A incidence among all phenotypes. Age-related resilience against physical trauma and NPDs likely underpins the lower incidence of NPDs in this particular phenotype. The highest risk of NPD-A was evident in the *older adults/extended recovery/supportive care* class. Previous research has shown that prolonged LOS, especially in the intensive care unit, is a risk factor for long-term psychiatric disorders. Recovering at home with support services and relocation to a nursing home is a challenging transition for older adults, often resulting in high rates of persistent depression and anxiety [35]. These environmental changes and age-related vulnerability coupled with the recovery from a head injury may exacerbate mental health symptoms with subsequent development of NPD-A. The *young-middle-aged males/extended recovery/rehabilitative care* phenotype was also associated with a high incidence of NPD-A. Interestingly, the severity of head injuries observed within this class was especially higher when compared to the other subgroups, suggesting that the extent of functional loss resulting from these injuries necessitated specialized rehabilitative care. Importantly, non-routine discharge to a supportive facility often implies that an individual has experienced functional and/or cognitive decline, which may serve as mediators in the emergence of NPDs. Individuals experiencing limitations in their ability to perform activities of daily living may develop feelings of helplessness and frustration, potentially leading to the development of NPDs. Similarly, cognitive decline after TBI may impede one’s ability to process and cope with stressors, increasing vulnerability to psychiatric disorders. Therefore, non-routine discharge to supportive facilities serves as an indicator of functional and/or cognitive loss, which in turn can play a mediating role in the development of NPDs. The current study and data sources did not allow for an analysis of how functional, cognitive, and social outcomes of head injury may be confounders, mediators, and/or modifiers of the association between TBI and NPDs. We acknowledge that a mediation analysis to quantify the extent to which the relationship between TBI and NPDs can be explained by one or more intermediate variables is critical in expanding our understanding of the complex causal pathway between head injury and NPDs.

We identified four distinct latent classes among TBI patients with pre-injury psychiatric history, each exhibiting a unique profile and highlighting the interaction between psychiatric burden and NPD-P incidence. The *young adult/low psychiatric burden* phenotype was characterized by the lowest burden of pre-existing psychiatric conditions and the lowest NPD-P incidence among all phenotypes. The reduced burden of psychiatric conditions is a likely explanation of the lower incidence of NPD-P in this particular phenotype. In contrast, the *psychiatric complexity/high comorbidity* class had the highest pre-injury psychiatric comorbidity levels and were discharged home post-injury. The high burden of psychiatric conditions coupled with the potential lack of screening and monitoring for NPDs in a home environment, may explain why this phenotype had the greatest NPD-P incidence among four classes.

Results of conditional logistic regression for each phenotype allowed for the comparison of TBI patients with their matched controls within homogeneous subgroups. We demonstrated that within each phenotype, patients with TBI are more likely to have NPD than matched controls, corresponding to meaningful differences in NPD risk when matched controls are considered. It is important to note that latent classes were derived using a broader set of indicators, including demographics and injury-related variables for patients with TBI. The matched controls, however, were matched to patients with TBI patients based on age, sex, comorbidities, and psychiatric conditions. This difference should be taken into account when interpreting the results of the conditional logistic regression with each phenotype.

Guidelines from the American College of Surgeons emphasize the importance of postinjury mental health disorder screening and intervention for trauma patients [36]. Triaging the risk of NPDs at the time of discharge from hospital may lead to better post-injury mental health outcomes and quality of life for patients with TBI. Our approach of identifying interpretable phenotypes with different risk of NPDs, and the accurate classification of individuals into these phenotypes, allows for a practical risk stratification approach of patients with TBI. The parameter estimates from the LCA model on the original data can be applied to calculate the posterior class membership probabilities for new patients. This process facilitates risk assessment by classifying new patients into one of the defined phenotypes.

### Strengths and limitations

This study has several strengths. The linked data sources provided detailed information for a population-based cohort, limiting selection and recall biases, while also ensuring robust statistical power. The variables used in our modelling approach are available in electronic health records, and therefore the insights from our analyses are applicable within similar settings. To assess the health burden of NPDs among individuals with and without TBI, a direct comparison of data between TBI cases and uninjured controls is essential. Hence, we employed a demographically similar uninjured cohort as the matched group for patients with TBI. This matching approach enables the exploration of differences in NPD prevalence between injured individuals and healthy controls.

There are limitations to be considered when interpreting the current findings. Our results are dependent on patient encounters documented in administrative databases, with the potential for under-reporting of diagnoses, lack of specificity in coding, and inaccuracies in designating diagnostic categories. Given that the current study used administrative health databases, we did not capture lifetime psychiatric history using structured clinical interviews. Our study may have potentially underestimated the true prevalence of pre-injury psychiatric history and its impact on the development of novel psychiatric disorders post-TBI using a lookback period of two years to ascertain pre-injury psychiatric burden. For patients with pre-existing psychiatric disorders, we were unable to determine whether these disorders worsened in severity based on ICD coding. Additionally, our study did not capture changes in life satisfaction, functional independence, and social participation, all of which are known to influence mental health functioning after injury [37, 38]. It is also important to acknowledge that while the control group was selected to match the TBI cohort demographically and did not have a recorded history of TBI or develop TBI during the two years of follow-up, some controls may have experienced head injuries which did not result in clinical encounters. Finally, the current study did not perform external validation of the identified phenotypes. To confirm the generalizability of these phenotypes, future research should apply the posterior membership probabilities derived from our main analysis to a new, independent sample of TBI patients. Nevertheless, the study findings provide a method to identify homogeneous subgroups of TBI patients in relation to NPDs, thereby facilitating both research and clinical applications. We demonstrate that LCA can distinguish clinically meaningful phenotypes using routinely collected variables. Using these standard variables may be a useful method to stratify patients into more homogeneous groups for enrollment into clinical trials, selection of pharmacological and non-pharmacological interventions, and prediction of clinical outcomes. From a clinical standpoint, the risk phenotypes would be useful for counselling patients and predicting their psychiatric rehabilitation needs.

## Conclusions

This study highlights the significant post-injury psychiatric burden experienced by survivors of TBI. Head-injured patients with and without a pre-injury psychiatric history have a significantly greater burden of NPDs compared with population-matched controls. TBI is a heterogenous disorder comprised of clinically distinguishable phenotypes with different patterns of NPD risk. Using this approach for risk stratification at the time of discharge from acute care may help guide early and targeted interventions for those individuals most at risk of developing NPDs following TBI.

### Electronic supplementary material

Below is the link to the electronic supplementary material.

Supplementary Material 1

Supplementary Material 2

## Data Availability

No datasets were generated or analysed during the current study.
