# Supplementary material for: Risk stratification of new-onset psychiatric disorders using clinically distinct traumatic brain injury phenotypes
Source: Arch Public Health. 2024 Aug 2;82:116. doi: 10.1186/s13690-024-01346-w (PMC11295665; doi:10.1186/s13690-024-01346-w)
Supplement: Supplementary file 1 — Supplementary Material 1 [file 13690_2024_1346_MOESM1_ESM.docx]

**Supplementary Data provided by Authors**

**Propensity Matched Analysis**

**Objective:** To determine the effect of TBI on the development of NPDs**.**

**Data elements:** Age (± 2 years) at index date, sex, Charlson Comorbidities Index (CCI) group: no comorbidities (0), mild (1- 2), moderate (3-4), and severe (≥5), calculated in two years lookback from index date, mental health diagnoses (ICD9:290-319, ICD10: F04-F99) categories.

**Methodology:**

Dataset Creation: The study cohort included patients diagnosed with TBI and controls matched on age, sex, past history of mental health disorders and comorbidities. Medical comorbidities were derived from CIHI-DAD and physician billing diagnostic codes.

Look-back Window

Observation Window

**Index Event Date**

Max Follow-up Date

The Observation Window terminated at a) End of the follow-up period (2 years from index date) b) Death of a person. The Lookback Window was defined as 2 years prior to index date to assess mental health diagnoses and CCI. The Maximum Follow-up Date was March 31,2021.

Study cohort

Case pool: All individuals diagnosed with TBI between April 1, 2002 - March 31, 2019, were provided from Nova Scotia Trauma Registry (NSTR). The index date for cases was the month/year of TBI as provided through the trauma registry data.

Control pool: All individuals from the insured patient registry who did not have a TBI diagnosis between April 1, 2002 - March 31, 2019, and follow-up period (see above). The insured patient registry comprises individual registered as a beneficiary of provincial healthcare services.

Eligibility of controls: Alive at index date, Nova Scotia resident.

Exclusion of controls: Missing values for all matching variables, TBI diagnosis for duration of study period (April 1, 2002-March 31, 2019) as well as follow up period (2 years after index date), or history of hospitalization 12 weeks prior to index date.

***Sample size calculation***

A sample size calculation was performed *a priori* to ensure the feasibility of this study using data available in the NSTR. The sample size calculation is based on a comparison of NPD rates among participants with TBI and controls from previous studies (Arif et al., 2021). Assuming an NPD rate of 53% in controls compared with an assumed NPD rate of 82% for those with TBI, an alpha value of 0.05, and a power of 0.9, a total of 165 patients were required.

**Latent Class Analysis**

**Objective:** To determine specific TBI phenotypes which differ with respect to demographics, injury variables, and pre-injury psychiatric conditions

We specify the following definitions:

*Phenotype:* a vector of distinguishing variable values/covariates that identify a specific entity, such as an individual.

*Phenotyping:* An unsupervised process of partitioning a heterogeneous population into homogeneous sub-populations, where each group has similar characteristics in terms of the predicting variables/covariates

**Data elements:** For those without psychiatric history, indicators included sex, categories of age, categories of CCI, injury mechanism, injury severity (maximum AIS Head), prolonged LOS, and discharge destination were used as indicators. For those with pre-injury psychiatric disorders, indicators included, past psychiatric conditions were added to the previously described indicators. See Supplementary Table 1 for details.

**Methodology:** LCA was conducted in R version 4.2 using the *poLCA* package. Relative fit was evaluated using the Bayesian Information Criterion (BIC), the sample-size adjusted BIC (SABIC), and Vuong-Lo-Mendell-Rubin adjusted likelihood ratio test (VLMR-LRT). The VLMR-LRT was calculated using the *tidyLPA* package in R. Lower values for BIC and SABIC indicate relatively better balance between parsimony and model fit. Emphasis was placed on BIC and VLMR-LRT given evidence showing their unique strength in identifying the ideal number of classes (Nylund et al., 2007). The VLMR-LRT compares the fit between two neighboring class models, where a nonsignificant *p* value indicates that the model with one fewer class provides a more parsimonious fit to the data.

After class enumeration, the classification accuracy of the models was evaluated using the matrix of average posterior probability of correct classification for each class. This classification metric describes the proportion in each class that is correctly classified in each of the classes, where high diagonal and low off-diagonal values reflect better classification. The assessment of empirical robustness was complemented by an emphasis on interpretability and theoretical utility during the process of model selection (Muthén, 2003). Final evaluation of models considered whether the final solution reflected distinct and interpretable groups. The classes were named in a way that best represented the most notable discoveries in the data, however, names are only for ease of presentation and do not necessarily reflect all differences between classes.
