# Supplementary material for: Risk stratification of new-onset psychiatric disorders using clinically distinct traumatic brain injury phenotypes
Source: Arch Public Health. 2024 Aug 2;82:116. doi: 10.1186/s13690-024-01346-w (PMC11295665; doi:10.1186/s13690-024-01346-w)
Supplement: Supplementary file 2 — Supplementary Material 2 [file 13690_2024_1346_MOESM2_ESM.docx]

**Supplementary Tables**

**Supplementary Table 1: Psychiatric Disorder History in Matched Cohort^1^**

| **Disorder** | **TBI** | **Control** |
| --- | --- | --- |
|  | n(%) | n(%) |
| Acute stress reaction | 61(5) | 245(5) |
| Adjustment disorder | 78(6) | 236(5) |
| Attention deficit disorders | 73(6) | 280(6) |
| Bipolar disorder | 82(7) | 235(5) |
| Conduct disorders | 12(1) | 45(1) |
| Dissociative, conversion, and factitious disorders | - | 15(0.3) |
| Eating disorders | 8(0.6) | 13(0.3) |
| Generalized anxiety disorder | 578(46) | 2248(47) |
| Learning disorders | 14(1) | 40(1) |
| Mood disorders | 375(30) | 1443(30) |
| Mental delay | - | 10(0.2) |
| Obsessive compulsive disorders | 7(0.6) | 28(0.6) |
| Organic disorders | 176(14) | 533(11) |
| Other neurotic disorders | 26(2) | 72(2) |
| Pain disorders | 21(2) | 72(2) |
| Personality disorders | 28(2) | 55(1) |
| PTSD | 9(1) | 23(1) |
| Sexual and gender identity disorders | 12(1) | 49(1) |
| Sleep disorders | 121(10) | 460(10) |
| Substance abuse disorder-alcohol | 145(12) | 247(5) |
| Substance abuse disorder-drugs | 178(14) | 796(17) |

1 ^1^Suppression of cell counts: If any cell is < 5, the value (and its corresponding percentage) is suppressed and indicated with dash (-). If a cell is < 5 and only one value is suppressed in a row or column, the next highest value in that row or column is also suppressed.

**Supplementary Table 2: Mean posterior probabilities of 4 class model (TBI cohort without pre-injury psychiatric conditions)**

|  | **Probability of assigned class** | | | |
| --- | --- | --- | --- | --- |
| **Assigned Class** | **1** | **2** | **3** | **4** |
| 1 | **0.84** | 0.06 | 0.11 | 0.01 |
| 2 | 0.06 | **0.92** | 0.00 | 0.07 |
| 3 | 0.11 | 0.00 | **0.86** | 0.03 |
| 4 | 0.01 | 0.07 | 0.03 | **0.90** |

**Supplementary Table 3: Mean posterior probabilities of 4 class model (TBI cohort with pre-injury psychiatric conditions)**

|  | **Probability of assigned class** | | | |
| --- | --- | --- | --- | --- |
| **Assigned Class** | **1** | **2** | **3** | **4** |
| 1 | **0.92** | 0.05 | 0.03 | 0.00 |
| 2 | 0.08 | **0.86** | 0.00 | 0.06 |
| 3 | 0.00 | 0.00 | **0.90** | 0.06 |
| 4 | 0.00 | 0.06 | 0.02 | **0.92** |
